# Mathematical modeling of disease dynamics in *SDHB*- and *SDHD*-related paraganglioma: Further step in understanding hereditary tumor differences and future therapeutic strategies

**DOI:** 10.1371/journal.pone.0201303

**Published:** 2018-08-14

**Authors:** Dominique Barbolosi, Joakim Crona, Raphaël Serre, Karel Pacak, David Taieb

**Affiliations:** 1 Aix-Marseille University, School of Pharmacy of Marseille, Simulation & Modelling: Adaptive Response for Therapeutics in Cancer (SMARTc), Marseille, France; 2 INSERM UMR U1068, CNRS UMR 7258, Aix-Marseille University, Cancer Research Center of Marseille, Marseille, France; 3 Department of Medical Sciences, Uppsala University, Uppsala, Sweden; 4 Section on Medical Neuroendocrinology, *Eunice Kennedy Shriver* National Institute of Child Health and Human Development, National Institutes of Health, Bethesda, United States of America; 5 Aix-Marseille University, Department of Nuclear Medicine, La Timone University Hospital, European Center for Research in Medical Imaging, Marseille, France; 2nd medical school of Charles University, CZECH REPUBLIC

## Abstract

Succinate dehydrogenase subunit B and D (*SDHB* and *SDHD*) mutations represent the most frequent cause of hereditary pheochromocytoma and paraganglioma (PPGL). Although truncation of the succinate dehydrogenase complex is thought to be the disease causing mechanism in both disorders, *SDHB* and *SDHD* patients exihibit different phenotypes. These phenotypic differences are currently unexplained by molecular genetics. The aim of this study is to compare disease dynamics in these two conditions via a Markov chain model based on 4 clinically-defined steady states. Our model corroborates at the population level phenotypic observations in *SDHB* and *SDHD* carriers and suggests potential explanations associated with the probabilities of disease maintenance and regression. In *SDHB-*related syndrome, PPGL maintenance seems to be reduced compared to *SDHD* (p = 0.04 vs 0.95) due to higher probability of tumor cell regression in *SDHB* vs *SDHD* (p = 0.87 vs 0.00). However, when *SDHB*-tumors give rise to metastases, metastatic cells are able to thrive with decreased probability of regression compared with *SDHD* counterparts (p = 0.17 vs 0.89). By constrast, almost all *SDHD* patients develop PGL (mainly head and neck) that persist throughout their lifetime. However, compared to *SDHB*, maintenance of metastatic lesions seems to be less effective for *SDHD* (p = 0.83 vs 0.11). These findings align with data suggesting that *SDHD*-related PPGL require less genetic events for tumor initiation and maintenance compared to those related to *SDHB*, but fail to initiate biology that promotes metastatic spread and metastatic cell survival in host tissues. By contrast, the higher number of genetic abnormalities required for tumor initiation and maintenance in *SDHB* PPGL result in a lower penetrance of PGL, but when cells give rise to metastases they are assumed to be better adapted to sustain survival. These proposed differences in disease progression dynamics between *SDHB* and *SDHD* diseases provide new cues for future exploration of *SDH*x PPGL behavior, offering considerations for future specific therapeutic and prevention strategies.

## Introduction

Pheochromocytomas and paragangliomas (PPGLs) are rare neuroendocrine tumors that arise either in head and neck parasympathetic paraganglia or paraaortic chromaffin tissue, which comprise sympathetic adrenal medulla and extra-adrenal paraganglia. Tumors that derive from either parasympathetic or sympathetic paraganglia are collectively named paragangliomas (PGLs) with the term pheochromocytoma (P) being restricted to adrenal PGL [1].

In up to 70% of cases, PPGL are associated with germline and somatic mutations in 15 well-characterized PPGL driver or fusion genes. The contribution to tumor initiation or progression of these disease driving genes is still not fully understood [2, 3]. This is well illustrated by the example of hereditary PGL syndromes. In 2000, Baysal *et al*. described the first PGL syndrome related to deficiency in succinate dehydrogenase (SDH) enzyme activity due to mutations in SDH subunit D (*SDHD*), part of mitochondrial complex II and the tricarboxylic acid (TCA) cycle [4]. This major discovery represents the first unequivocal genetic link between a mitochondrial defect and PPGL development. Association between the TCA cycle and PPGL was later confirmed by the identification of mutations in other genes encoding subunits B [5], C [6], and A [7] of the SDH complex or its flavination factor (*SDHAF2*) [8] and more recently, mutations in *fumarate hydratase* [9] and *malate dehydrogenase type 2* [10].

These genes are related to the TCA cycle and they are considered tumor supressors with biallelic inactivation of the healthy allele through a somatic event in paraganglial cells. This results in the accumulation of succinate which has pro-oncogenic effects via intracellular and extracellular (« hormone » like) actions and tumorigenesis [11, 12]. Activation of the hypoxia-inducible factor (HIF) signaling pathway despite normal oxygen supply (also called pseudohypoxia), as well as DNA and histone demethylases inhibition resulting in a hypermethylated genome, are two processes that were uniquely identified to contribute to transformation of a paraganglial cell into PPGL [12, 13].

Currently, *SDHB* and *SDHD* mutations represent the most frequent cause of hereditary PPGLs associated with TCA defects. In *SDHD*-patients, PGLs in the head and neck region and anterior/medium mediastinum can be found in 85% of cases wheras less than 5% have abdominal PGL. HNPGL and abdominal PGL coexist in 10% of cases. *SDHB*-linked PGL syndrome is characterized by a high rate of abdominal PGL (70–80%). HNPGLs occur in 20–30% of cases. The coexistence of HNPGL and abdominal PGL is rare (<3%). It is also notable that multifocality mainly occurs in *SDHD* cases (at least 60% vs 20% for *SDHB*) [14]. PGLs with underlying *SDHB* mutations are associated with a higher risk of aggressive behavior, development of metastatic disease, and ultimately, death [15]. Overall, the risk of metastatic disease in *SDHB* mutation-associated tumors has been estimated to be 30% vs <5% for *SDHD*. The transmission of disease is also different. Although *SDHD* and *SDHB* are both autosomal dominant diseases, the penetrance of *SDHD*-related PPGL is modulated by maternal imprinting. Overall disease penetrance of *SDHB* and *SDHD* diseases is dependent on the use of high sensitive imaging investigations in the work-up of non probands but also the molecular severity of the variants. *SDHD*-related mutations (paternally inherited) have very high penetrance (90–100%), in contrast to *SDHB* ones that have an estimated penetrance of only 20–40% [16–19]. A lower *SDHD* disease penetrance may be observed in studies that included low severity mutations [20].

As there is no biological experimental system that successfully replicates the human phenotype, we sought to understand the phenotypic heterogenity of TCA cycle-related PGL syndromes by computational modeling. Our results provide novel insights on potential causes of differential dymanics of PPGL tumorigenesis in *SDH* carriers.

## Results and discussion

We have chosen to use a Markov chain model because it is a well-accepted probabilistic approach for modeling a change between a fixed number of disease states over time. For more than twenty years, Markov models have been used in various areas of medical research, such as cost-effectiveness studies [21], epidemiologic analysis [22, 23] or genome research [24]. Our Markov model simulates transitions between various clinically defined states.

### In *SDHB*-related PPGL syndrome

**Fig 1:**
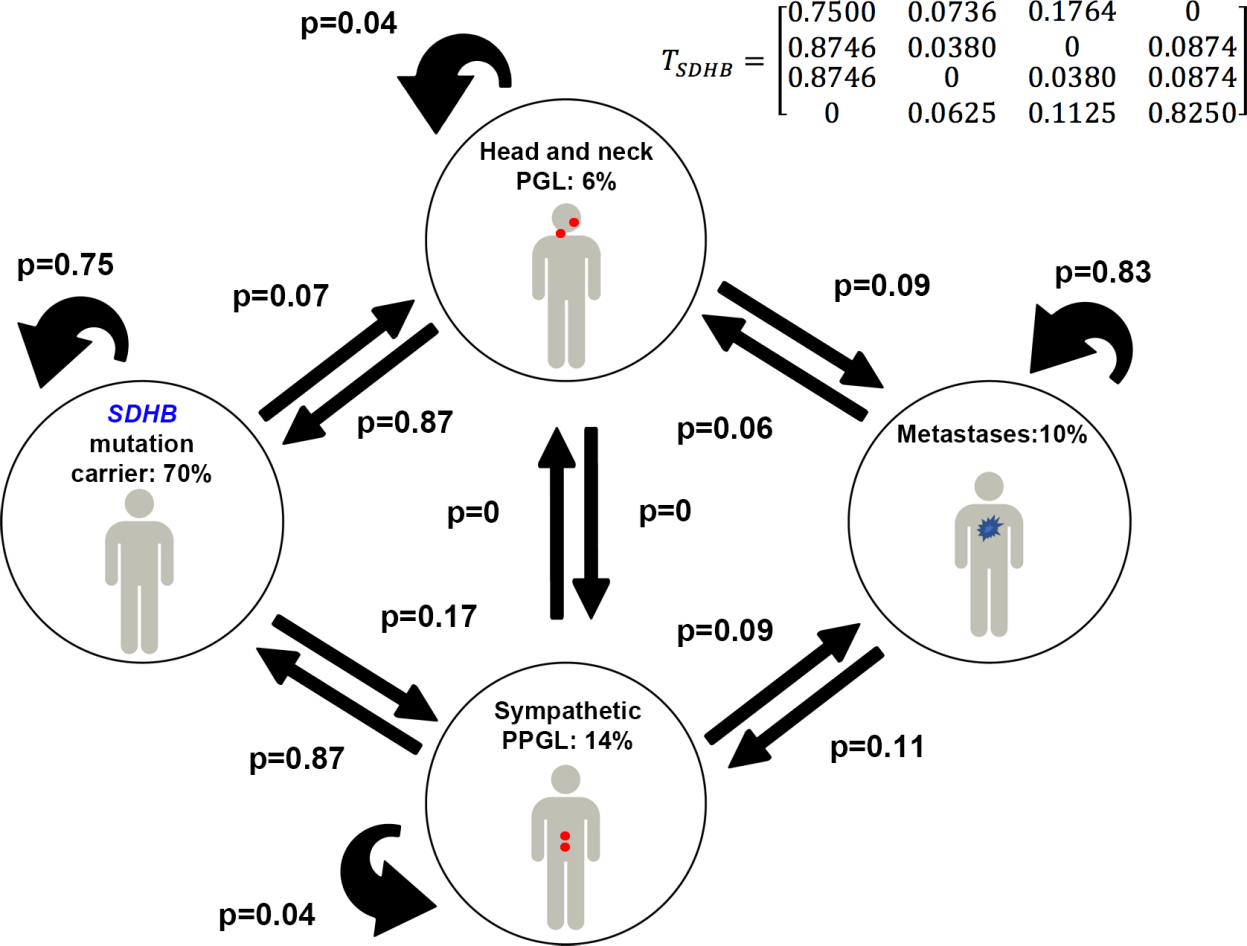
Clinical states and transitions in *SDHB*-mutation carriers. Transition probabilies are provided in the T_sdhb_ matrix, with 4 rows and 4 columns, upper right. Numerical values for steady states and transition probabilities are displayed here and also given in Table 1 and Table 2. For example, p_12_ is the probability of moving from state 1 to state 2 and *p*_*11*_ is the probability to stay in this state 1.

For modeling disease dynamics in *SDHB* PPGL, the following parameters, derived from clinical studies were used: P^b^_obs_ = [0.70, 0.06, 0.14, 0.10] (where the superscript “b” stands for *SDHB*); i.e: 70% will remain without disease corresponding to 30% penetrance, and among patients with active disease: 6% HNPGL and 14% sympathetic PPGL, 10% metastatic disease). For the particular value p_11_ = 0.75, a transition matrix T_sdhb_ was estimated and is shown in Fig 1 (in this matrix, for i = 1..4, j = 1..4, the number in row i and column j is transition probability *p_ij_*). This choice is p_11_ is discussed in the Sensitivity Analysis section.

The model allowed us to simulate PPGL initiation and spontaneous regression of tumors and demonstrate how these two processes can contribute to the disease phenotype. In this case, the asymptotic probability distribution P*_sdhb_ (at steady-state, which gives the probabilities to be in one of the four states in the course of life) was: P*_sdhb_ = [0.7, 0.06, 0.14, 0.10], describing exactly the clinical picture of the disease P^b^_obs._ Our principal findings show that for *SDHB* sub-group, the stabilization into a clinical state with the development of PPGL is a process driven by high rate of regression to a state without a (clinically detectable) tumor (low p_21_ and p_31_). However, when PPGL develops, it easily gives rise to metastasis and metastatic PPGL sustains survival (high p_44_).

### In *SDHD*-related PGL syndrome

**Fig 2:**
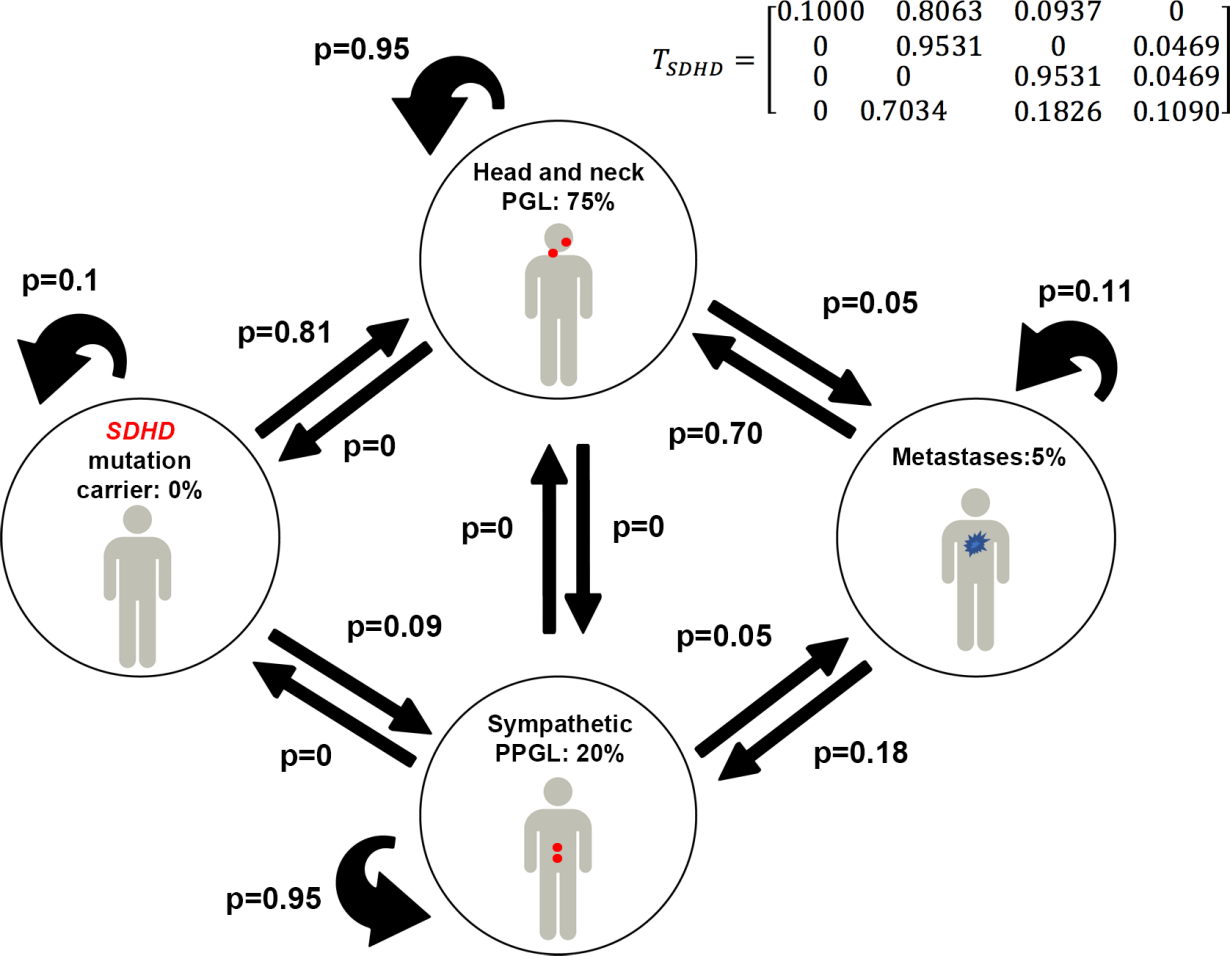
Clinical states and transitions in *SDHD*-mutation carriers. Only the situation where the pathogenic variant is inherited from the father is considered. Transition probabilies are provided in the T_sdhd_ matrix. Transition probabilities are represented in a 4–4 matrix (with 4 rows and 4 columns, upper right). Numerical values for steady states and transition probabilities are displayed here and also given in Table 1 and Table 2.

A first model was parameterized to simulate *SDHD* disease dynamics with the following set of P^d^_obsv_ = [0.00, 0.75, 0.20, 0.05], where the superscript “d” stands for SDHD (i.e, 100% penetrance if the mutation is inherited from the father, 75% HNPGL and 20% sympathetic PPGL, 5% metastatic disease). The T_sdhd_ matrix is shown in Fig 2 with the following probabilities at steady state: P*_sdhd_ = [0.00, 0.75, 0.20, 0.05]. These results accurately describe the observed probabilities.

The observed *SDHD* PPGL steady-state values were characterized by high values of p_12_, p_11_, p_22_ and p_43_ and low values of p_21_ and p_31_. Therefore, it seems that *SDHD* PPGL (mainly those from parasympathetic paraganglia) develop more easily than *SDHB* ones and persist throughout the life (high p_12_) of a patient. Furthermore, although *SDHD* primary PPGL can transition into metastatic disease, it mostly fails to develop a stable advanced disease state and are therefore not diagnosed as metastatic (low p_44_).

### Differences between *SDHB* and *SDHD*

Our model corroborates clinical observations by showing that *SDHB* and *SDHD* carriers have different dynamics in PPGL occurrence, regression, and progression thus, providing new insights into the understanding of these tumors. Tables 1 and 2 summarizes the main differences between *SDHB* and *SDHD* regarding the probabilities for tumor maintenance or regression. First, tumor maintenance is reduced in *SDHB* patients (p_22 and_ p_33 =_ 0.04) compared to *SDHD* patients (p_22_ and p_33 =_ 0.95). Second, the probability of staying in metastatic state is, however, higher for *SDHB* than *SDHD* patients (p_44 =_ 0.83 vs 0.11).

**Table 1:** Estimated percentages at different clinically-defined steady states in *SDHB* and *SDHD*-related PPGL.

	*SDHB*	*SDHD*
Patients without tumor	70% (60%-80%)	0% (0%-10%)
HNPGL	6% (3%-8%)	75% (61%-80%)
Sympathetic PPGL	14% (8%-18%)	20% (12%-28%)
Metastases	10% (6%-13%)	5% (3%-7%)
Total	100%	100%

**Table 2:** Differences in estimated transition probabilities into the different clinically-defined steady states in *SDHB* and *SDHD*-related PPGL.

	*SDHB*	*SDHD*
**HNPGL**		
p to maintain	0.04 (0.01–0.25)	0.95 (0.85–0.96)
p to regress	0.87 (0.61–0.99)	0.00 (0.00–0.10)
**Sympathetic PPGL**		
p to maintain	0.04 (0.01–0.25)	0.95 (0.85–0.96)
p to regress	0.87 (0.61–0.99)	0.00 (0.00–0.10)
**Metastases**		
p to maintain	0.83 (0.64–0.99)	0.11 (0.10–0.25)
p to regress if originate from HNPGL	0.06 (0.01–0.13)	0.70 (0.43–0.71)
p to regress if originate from sympathetic PPGL	0.11 (0.01–0.23)	0.18 (0.15–0.31)
p to regress (from HNPGL)/p to maintain (ratio)	0.07 (0.01–0.19)	6.36 (1.75–6.78)
p to regress (from sympathetic PPGL)/p to maintain (ratio)	0.13 (0.01–0.36)	1.63 (0.70–2.19)

*SDHB* data provided for p_11_ = 0.75; *SDHD* data provided for p_11_ = 0.10; the 95% confidence intervals are provided within parenthesis.

The sensitivity analysis shows robustness of the model with respect to reasonable variations in the 4 percentages of disease, which supports the validity of the modeling method. One parameter (p_11_) had to be fixed prior to the estimation procedure, which was unavoidable to ensure that the number of free-parameters to estimate was equal to 4, the number of observations, as described in the Sensitivity Analysis section.

Our discrete-time Markov chains model simulates transition into clinically-defined steady states and describes qualitative differences in disease dynamics between the 2 syndromes. However, unlike continuous-time Markov chains, it does not provide informations of disease dynamics over time.

Our model does not define the mechanisms underlying transition probabilities that could in theory reflect any biological mechanism. Nevertheless, through an integration of current knowledge of PPGL tumorigenesis, we attempted to integrate our findings into two major knolwedges related to *SDHB-* and *SDHD*-PPGL tumorigenesis: a genetic background, here the presence of *SDHB* or *SDHD* mutations [25], and embryological development from either sympathetic of parasympathetic paraganglia [26].

Here, genetics stipulates that biallelic inactivation of *SDHB* or *SDHD* loci is not enough to cause PPGL. It is only through deregulation of additional genes, cellular, epigenetic, microenvironmental, and other events that PPGL can be formed. Due to location in the genome, *SDHD* could require fewer genetic hits than *SDHB* to form PPGL. *SDHD* as well as *SDHAF2*-related PPGL are characterized by a specific loss of maternal chromosome 11 [27, 28], a finding which is consistent with paternal transmission of the diseases (both genes being located in chromosome 11). This is also observed in *VHL*-related pheochromocytoma [29], suggesting the potential role of several maternally expressed genes in tumorigenesis. By contrast, *SDHB* PPGL are characterized by a lower frequency of chromosome 11 loss (31% of cases) with a more complex pattern and a greater genomic instability compared to *SDH* with gains and losses confined to other chromosomes. It is, therefore, possible that chromosome 11p loss is necessary and sufficient to trigger *SDHD* and *SDHAF2* tumorigenesis, whereas *SDHB* tumors require more complex changes with amplification or deletion of multiple driver genes located on different chromosomes, especially 1p. Assuming a constant rate of genetic instability, this could be in agreement with differences in penetrance of the *SDHD* and *SDHB* disease. A second assumption is that additional genetic events contribute to the transformation of PPGL cells into a biology that favours metastatic spread [2, 29]. For *SDHB-*related PPGL, these would have accumulated more genetic events at the time of primary tumor development, therefore having a higher probability to aquire such metastasis promoting genetic events. This speculation potentially explains the very low probabilities of *SDHB* PPGL compared to *SDHD* to return from state 4 (metastatic disease) to state 2 (p_42_) and 3 (p_43_) presenting with no metastasis. This is reflected by the probability of staying in state 4 as being very high for *SDHB* and very low for *SDHD* (p_44_). Interestingly, tumor maintenance is reduced in *SDHB* patients (p_22_ and p_33 =_ 0.04) due to a high probability for tumor regression (p_21_ and p_31 =_ 0.87). This dynamic is inverted in *SDHD*-related PPGL, which are characterized by a high probability for tumor maintenance (p_22_ and p_33 =_ 0.95). These opposite pathways (tumor formation/regression) suggest that genetic abnormalities present in *SDHB* tumors could involve genes that play a role in execution of gene programming and signaling that control G1-S and G2-M cell cycle checkpoints and death receptor/apoptosis events. A precise threshold of these proteins could be required for maintaining a specific tumor state, where there is a switch from proliferation to a state of proliferative arrest and apoptosis. This property has been illustrated for MYC [30]. We acknowledge that this theory is still only supported by indirect evidence from genetic data that also fails to explain the absence of somatic biallelic *SDH* inactivation.

The metastatic capability of *SDHB*-deficient tumor cells could also be related to intrinsic capacity of sympathetic nervous system cells to develop metastasis. By contrast in *SDHD* mutated patients, embryological development could result in the abnormal foundation of a parasympathetic nervous system that lacks an intrinsic capacity to form metastasis. This model remains theoretically plausible, but is not currently supported by experimental evidence.

In light of what is currently known of PPGL tumorigenesis, we suggest that our results could be interpreted as follows: in *SDHD*, a limited number of genetic abnormalities seem effective for tumor initiation and maintenance (high p_12_), but fail to initiate a biology that promotes metastatic spread and cell survival in host tissues (low p_44_). By contrast, the higher number of genetic abnormalities required for tumor initiation and maintenance in *SDHB*-related PPGL result in a lower penetrance of PGL (low p_21_ and p_31_), but when cells give rise to a tumor followed by metastases, they seem to be more adapted to sustain survival (high p_44_).

These findings suggest that therapeutic strategies against *SDHB* should be prioritized for killing cells at early stages of metastatic spread, either with no detected tumors by imaging (e.g. adjuvant systemic therapies) or detectable tumors (e.g. radio- or immunotherapies). By constrast, for *SDHD*, a major goal would be to prevent mechanisms involved in tumor development and maintenance (prevention of second somatic hit via antioxydants or drugs that reduce endogenous mutations, limiting of exposure to ionizing radiations) since metastatic cells are more instable. The model could also be used to test the disease dynamics following a particular treatment. These findings would ultimately have to be supported by various clinical trials and interventions in patients or experimental models.

## Methods

### Model design

TCA-related hereditary PPGL occurrence, progression, or regression are assumed to be a population-level random processes in which it is currently only known that metastasis occurs more frequently from large and sympathetic primary PPGLs. Such assumptions and knowledge are well suited to be simulated with a Markov chain model that links together a series of stochastically generated events that, over time, result in a clinically significant steady state.

Only 4 PPGL-related disease states were considered:

○ State 1: no tumor present
○ State 2: presence of head and neck paraganglioma (PGL)
○ State 3: presence of pheochromocytoma or sympathetic PGL
○ State 4: presence of metastases (i.e., malignancy).

The transition probabilities are denoted by *p*_*ij*_ and *p*_*ii*_, where *p*_*ij*_ is the probability of moving from state *i* to state *j* and *p*_*ii*_ is the probability to stay in this state *i*.

**Fig 3:**
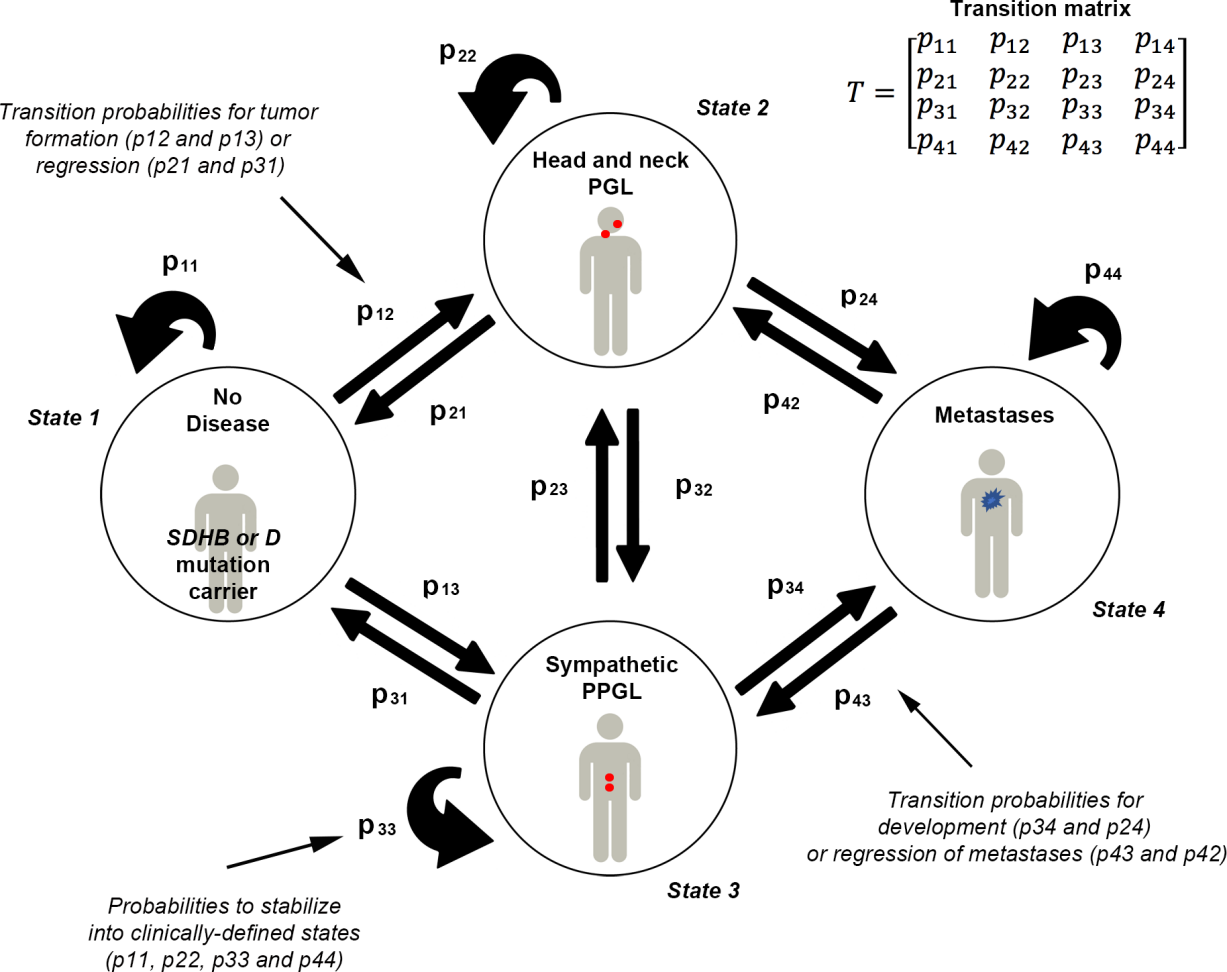
Disease scenarios and rules of disease dynamics. Four clinical states and 16 transition probabilities between states are represented (using Markov chains). Population-level transition probabilities are represented in the 4–4 matrix *T* (i.e with 4 rows and 4 columns, upper right). The transition probabilities are denoted by *p*_*ij*_ and *p*_*ii*_, where *p*_*ij*_ is the probability of moving from state *i* to state *j* and *p*_*ii*_ is the probability to stay in this state *i*. Numerical values for transition probabilities are given in Table 1. According to international nomenclature of stochastic matrix, the row vector of the Markov chains has been written in order to meet the following criteria: non negative coefficients and the sum of each row equal to 1.

Usually, for a Markov chain with 4 states, the transition probabilities are represented in a 4–4 matrix *T* (i.e with 4 rows and 4 columns, Fig 3).

For *i*,*j = 1*,*2*,*3*,*4*, the coefficients p_ij_ of the 4–4 transition matrix T can be defined as follows for any step *n*:

1. p_11_ is the probability of an *SDHB* or *SDHD* mutation carrier not having any tumor (state 1) to stay in this state
2. p_12_ is the probability of a patient moving from state 1 to state 2 and developing HNPGL, which could be microscopic in size
3. p_21_ is the probability of a patient with HNPGL to return to state 1 from state 2
4. p_13_ is the probability of moving from state 1 to state 3 and developing sympathetic PPGL, which could be microscopic in size
5. p_31_ is the probability of a patient with sympathetic PPGL to return to state 1 from state 3
6. p_24_, p_34_ are the probabilities of a PPGL (state 2 or 3, respectively in HNPGL or sympathetic PPGL) to develop metastasis
7. p_42_, p_43_ are the probabilities of returning to state 2 and 3 due to death of metastatic cell(s) from the state 4
8. p_23_, p_32_ are the probabilities of moving from HNPGL to sympathetic PPGL and *vice versa*

These four states reflect potential clinical scenarios in a given population of patients. Transition between these states will occur until stabilization of the population into each of the four states. Thus, the steady (“final”) state represents the health status of a group of patients observed/diagnosed by a physician. However, the chain of intermediary states is not observable, because it is a chain of hidden events, driven by randomness that eventually results in the observable health condition of a group. The probability for a group of patients to be stable in any of these four states is based on clinical evaluations and here, by studies related to PPGLs. Changes within one state are called transitions and the model was designed to allow for a change of state in both directions i.e., both tumor development or its regression. Underlying this design is the assumption that PPGL could regress or metastasize through an intermediate step (PPGL).

Furthemore, the existence of transitions back from the metastatic state to one of the two non-metastatic states (HNPGL or sympathetic PPGL) should not be misunderstood as a spontaneous regression from a diagnosed state of metastasis to a benign tumor state. Instead, the model assumes a scenario where not all metastases survive and only a small proportion of them establish into actual metastatic disease. Hence, it is assumed that the chain of intermediary states will always contain a (random) number of transient metastatic steps before the population of patients will stabilize into the four states at time of clinical intervention.

### Model estimation

Transition probabilities, which are “invisible” for clinicians, were estimated in order to fit with the limits P* corresponding to the phenotype observed in *SDHB* and *SDHD* PPGL syndromes: P^b^_obvs_ or P^d^_obvs_. Hence, the transition probabilities p_ij_ selected will be those that produce the theorical value P*[i], to be in state i throughout life, which is closest to observed value P_obvs_[i]. In others words, mathematically, these transition probabilities were estimated by using the method of the mean least squares, which select the p_ij,_ which minimizes the quantity: (P*[1]—P_obvs_[1])^2^ +(P*[2]—P_obvs_[2])^2^ +(P*[3]—P_obvs_[3])^2^ +(P*[4]—P_obvs_[4])^2^.

The following assuptions were made in calculating transition probabilities:

1. the transition probability *p*_11_ was fixed; this choice being discussed below;
2. to match the number of free parameters with the number of steady-states, it was assumed that transition probabilities do not depend on tumor location (i.e. head and neck PGL vs. sympathetic PGL): p_21_ = p_31_ and p_22_ = p_33_;
3. since the coexistence of head and neck PGL and sympathetic PPGL is very rare condition, the transition probabilities p_23_ and p_32_ were fixed at zero;

These assumptions have allowed reduction of free-parameters to 4: (p_21_, p_22_, p_24_, p_44_), the other parameters being either fixed (p_11_ = 0.75 for *SDHB* and p_11_ = 0.10 for *SDHD*), or set at zero (p_23_ = 0, p_32_ = 0), or constrained by the two assumed relationships (p_21_ = p_31_), (p_22_ = p_33_), and, obviously, by the fact that the transition probabilities from one state must sum to one. Then the 4 free-parameters were estimated to fit the 4 clinically-defined steady states.

The estimation procedure was done with *lsqnonlin* of the MATLAB software, which implements a constrained non-linear least-square minimization routine.

### Sensitivity analysis

Since PPGL penetrance and the proportion of patients with either HNPGL, sympathetic PGL, or metastatic disease are *uncertain* numbers, it is important to assess the stability of transition probabilities if one moves observed probabilities within a range of realistic values. A model that would produce large swings of transition probabilities for small changes in observed probabilities of disease should be rejected for lack of stability (or robustness). Therefore, the stability of the model has been assessed by running many computations against different probabilities of stable states drawn at random: the 95% confidence interval for *SDHB* at state 1 was set to 60–80% (corresponding to a 20–40% penetrance, which corresponds to the established penetrance range) and to 0–10% for *SDHD* (corresponding to a 90–100% penetrance already cited). The uncertainty on the proportion of patients in states (2, 3) has been described with two independant Gaussian laws of distribution with a relative standard deviation of 20% (the choice of 20% is arbitrary, but it is conservative as it is relatively large and challenges the stabilitiy of the model). The proportion of patients in a metastatic state was logically chosen so that the sum of the four probabilities would be one. For each random observation generated by this procedure, a check was done to verify that the four probabilities were in [0,1], then the model was solved with the least-square method already described; the quality of the fit was verified by checking residuals and the output of the solving algorithm and the probabilities of transition were stored. Eventually (after 500 runs), the 95% confidence intervals on the transition probabilities were extracted.

The robustness of the estimation procedure has been evaluated for various values of p_11_, since this parameter has been fixed in the model. Simulations showed a good fit between clinical observations and model predictions in a wide range of p_11_ values: [70%, 99%] for *SDHB* and [1%, 99%] for *SDHD*. In *SDHB*-related PPGL, the quality of fit decreased rapidly for values of p_11_ below 0.70. However, the results remain qualitatively unchanged and lead to the same biological interpretations regarding transition probabilities. For simplicity, it was decided to provide the estimated results of transition probabilities for p_11_ = 0.75 for *SDHB* and p_11_ = 0.10 for *SDHD*, since these values are linked to the overall disease penetrance of both *SDHB* and *SDHD* diseases.
